# Antiplatelet agents aspirin and dipyridamole, and the risk of different carcinoma in patients with type 2 diabetes mellitus: A Taiwan retrospective cohort study

**DOI:** 10.1097/MD.0000000000030468

**Published:** 2022-09-16

**Authors:** Hsing-Yi Huang, Tz-Wen Lu, Hsiu-Ling Liang, Wei-Hao Hsu, Ya-Wen Sung, Mei-Yueh Lee

**Affiliations:** a Division of Endocrinology and Metabolism, Department of Internal Medicine, Kaohsiung Medical University Hospital, Kaohsiung Medical University, Kaohsiung, Taiwan; b Department of Nursing, Kaohsiung Medical University Hospital, Kaohsiung, Taiwan; c Department of Internal Medicine, Kaohsiung Municipal Siaogang Hospital, Kaohsiung Medical University, Kaohsiung, Taiwan; d Faculty of Medicine, College of Medicine, Kaohsiung Medical University, Kaohsiung, Taiwan.

**Keywords:** aspirin and dipyridamole, cancer, diabetes mellitus, liver cancer

## Abstract

Studies have shown aspirin decreases the risk of some cancers. However, the evidence reported the association between aspirin and cancer risk in the diabetic population. In this study, we investigate whether aspirin and dipyridamole decrease the risk of cancer in patients with type 2 diabetes. A total of 5308 patients with type 2 diabetes were identified by the National Health Insurance from 1998 to 2000 and followed up until 2013. The demographic characteristics among nondipyridamole nor aspirin, aspirin, and dipyridamole users were analyzed by using the χ(2) test. Cox proportional hazard regression models were used to determine the independent effects of no aspirin nor dipyridamole, aspirin, and dipyridamole users on the risk of different cancer. After adjustment with multiple covariates, both low and high doses of aspirin and dipyridamole decrease liver cancer with risk ratios of 0.56 (95% CI, 0.37–0.83), 0.14 (95% CI, 0.05–0.39), 0.61 (95% CI, 0.38–0.99), and 0.28 (95% CI, 0.12–0.66), respectively. Both low and high doses of aspirin decrease any types of cancer with risk ratios of 0.79 (95% CI, 0.64–0.98) and 0.49 (95% CI, 0.34–0.70), respectively. Therefore, we conclude aspirin may decrease any types of cancer and liver cancer, and dipyridamole may decrease the risk of liver cancer in patients with type 2 diabetes.

## 1. Introduction

According to a World Health Organization report in 2014, 422 million people had diabetes worldwide,^[1]^ and this number was expected to rise to 642 million in those aged 20 to 79 years by 2040.^[2]^ In Taiwan, 1228,800 adults aged 20 to 79 years had diabetes in 2019, and the prevalence of diabetes was 6.6% in 2017.^[3]^ In addition, the total number of people with diabetes mellitus increased by 66% to approximately 875,000 patients from 2005 to 2014.^[4]^

In 2017, type 2 diabetes was the 10th leading cause of death globally, with a mortality rate of 13.2 per 100,000 persons.^[5]^ Cancer was the second cause of death worldwide in 2017, and every 6th death is due to cancer.^[6]^ In Taiwan, cancer and diabetes were the 1st and 5th leading causes of death in 2018, accounting for 28.2% and 5.4% of all cases of mortality, respectively.^[7]^ Both diabetes and cancer are multifactorial and chronic diseases. Many factors have been associated with the incidence of cancer and diabetes, including age, sex, ethnicity, metabolic syndrome, overweight, obesity, dietary intake, physical activity, smoking, and alcohol consumption.^[8]^ In addition, type 2 diabetes has been reported to have a higher risk of cancers including liver, pancreas, colon, bladder, endometrial, breast cancers, and non-Hodgkin’s lymphoma.^[9]^

Diabetes has been reported to modify carcinogenesis through hyperinsulinemia, hyperglycemia, and chronic inflammation.^[8]^ Insulin may have direct effects on cancer cells, and hyperinsulinemia may have an indirect effect on carcinogenesis promotion through insulin-like growth factor 1 (IGF-1). Hyperinsulinemia leads to an increase in the bioactivity of IGF-1 by inhibiting IGF binding protein-1.^[10]^ Mitogenic and antiapoptotic activity of mature IGF-1, as well as different transcripts and precursor IGF-1 peptides, qualify IGF-1 to the group of growth factors implicated in the initiation and progression of various cancers.^[11]^ High glucose serves as a subordinate plausible explanation of carcinogenesis. Hyperglycemia may exert direct and indirect effects upon cancer cells to promote proliferation.^[10]^ A systematic review and meta-analysis of 13 case-control and 13 cohort studies performed in 2005 reported that individuals with diabetes had a 2.5-fold higher risk of hepatocellular carcinoma compared to controls. Moreover, this strong association between hepatocellular carcinoma and diabetes was independent of alcohol consumption and hepatitis viral infection.^[12]^ Liver cells are exposed to higher concentrations of insulin compared to other tissues due to portal circulation, and this effect is worsened in patients with type 2 diabetes and insulin-resistant hyperinsulinemia.

Aspirin has the unique capability of inhibiting both isoforms of cyclooxygenase (COX-1 and COX-2) to reduce the synthesis of eicosanoids, which will be subsequently converted via cell-specific synthases into biologically active prostaglandins (PGE2, PGF2a, PGI2) and thromboxane A2. Several studies have reported that aspirin can decrease the risk of various cancers, including leukemia and esophageal, liver, pancreas, gastric, colon, and lung cancers.^[13]^ In addition, daily low-dose aspirin treatment was associated with a 20% decrease in the overall incidence of cancer 5 years after initiating treatment and a 30% decrease >5 years after treatment in 6 primary prevention trials.^[14]^ Patients with diabetes are at a greater risk of cardiovascular disease and related complications than people without diabetes. Aspirin blocks the synthesis of thromboxane and it is given to prevent stroke. Of particular importance, to both its cardiovascular and cancer indications, is the drug’s unique ability to irreversibly inhibit platelet COX-1 (via acylation of serine 530), preventing platelet activation over the remaining lifetime of the affected platelets. Aspirin also shares the ability with other nonsteroidal anti-inflammatory drugs to nonspecifically inhibit COX-2, which is overexpressed in many cancers.^[15]^ Because PGE2 promotes tumor proliferation, differentiation, and angiogenesis, it is widely considered that the anticancer effect of aspirin and related nonsteroidal anti-inflammatory drugs resides primarily in their COX-2–inhibitory activity, which is supported by preclinical and clinical evidence that COX-2–selective inhibitors (coxib) possess chemopreventive activity. However, no study has yet investigated the effect of aspirin to prevent cancer in patients with diabetes.

Dipyridamole is a pyridopyrimidine derivative that acts as a phosphodiesterase inhibitor to modestly reduce platelet function,^[16,17]^ and it has also been shown to be a vasodilator via endothelial activity.^[16,18]^ Dipyridamole has been shown to have potential benefits for patients with diabetes to prevent diabetic nephropathy.^[19^ The main indication for dipyridamole is stroke prevention. Emerging evidence, in vitro and in vivo, has shown an anticancer effect of dipyridamole via its antiviral activity, immunoregulation, autophagic flux blockade, antiproliferative activity, inhibiting tumor cell metastasis, and it enhancing the cytotoxicity of anticancer drugs.^[20–22]^ However, as with aspirin, there is no indication currently for the use of dipyridamole in cancer prevention for patients with diabetes.

Taken together, these findings show that diabetes mellitus has a strong relation with cancer and that aspirin and dipyridamole may have protective effects against the occurrence of cancer, but that these effects have not been investigated in patients with diabetes. Therefore, this study wants to investigate whether aspirin and dipyridamole can decrease the risk of cancer in patients with type 2 diabetes.

## 2. Materials and Methods

### 2.1. Study population

The National Health Insurance (NHI) program was launched in 1995 to provide healthcare and access to medical services for all citizens of Taiwan. As of the end of 2018, 23,948,000 people were registered in the NHI program and the coverage rate was more than 99% of the whole population.^[23]^

The National Health Insurance Research Database (NHIRD) contains the original claims data of the NHI, including diagnoses, prescriptions, and inpatient and outpatient claims data. Diagnoses in the NHIRD are coded using the International Classification of Diseases, Ninth Revision, and Clinical Modification (ICD-9-CM) codes. All patients who were diagnosed with type 2 diabetes mellitus (ICD-9-CM codes 250.x0 and 250.x2) and were prescribed with 2 or more times antihyperglycemic medications during outpatient visits from 1998 to 2000 were enrolled in this study. We excluded patients with type 1 diabetes mellitus (ICD-9-CM code 2501), those under 18 years of age at entry, those who had ever used aspirin/dipyridamole, and those with a cancer history. In order to ensure that we only enrolled newly diagnosed cancer cases, we excluded patients with any type of cancer (ICD-9-CM codes 140-208) from our diabetic patient groups. In Taiwan, all patients with a new diagnosis of cancer can apply for a catastrophic illness card through the NHI program. Their medical records will then be recorded directly onto their NHI card when they receive medical care associated with the related malignancy during admission or an outpatient department visit. To ensure the accuracy of the cancer diagnosis, we only selected the patients who used catastrophic illness cards at least twice during hospital admissions. In addition, to identify the patients who had and had not used aspirin/dipyridamole in the inpatient claims database, we separated them into 3 groups as follows: aspirin use only, dipyridamole use only, no aspirin nor dipyridamole use. The enrollment period was from January 1, 1998 to December 31, 2000, and the patients were followed until December 31, 2013. The final study cohort included 5308 patients.

Data on the occurrence of cancer during the follow-up period from January 1, 2001 to December 31, 2013 were obtained from the NHIRD. The types of cancer we discussed in this study included liver, colon, lung and bladder cancer, and lymphoma. The patients’ demographic and medical data included age, sex, renal disease, severe liver disease, a history of acute myocardial infarction, congestive heart failure, cerebral vascular events, peripheral vascular disease, paraplegia, dementia, pulmonary disease, peptic ulcer, liver disease, nephropathy, retinopathy, neuropathy, connective tissue disorders, metastatic cancer, and human immunodeficiency virus. Data on the use of medications including antihypertension medications like angiotensin II receptor blockers, angiotensin-converting enzyme inhibitors, calcium channel blockers, beta-blockers, diuretics, hydralazine, alfa-blockers, and other antihypertension drugs were also recorded. Antihyperglycemic medications like insulin, metformin, sulfonylureas, meglitinides, acarbose, dipeptidyl peptidase 4 inhibitors, glucagon-like peptide 1 agonists, and thiazolidinediones were also included.

### 2.2. Study endpoint

The study endpoint was a new diagnosis of malignancy from January 1, 2001 to December 31, 2013. In this study, we focused on the types of malignancy commonly associated with type 2 diabetes mellitus, including liver, colon, lung and bladder cancer, and lymphoma.

### 2.3. Statistical analysis

Data were expressed as a percentage or mean ± SD. The chi-square test was used to compare clinical characteristics between categorical variables, including the patients with diabetes who had taken aspirin, dipyridamole, or neither. Comparisons among study groups were conducted using a one-way analysis of variance. We used COX regression analysis to calculate hazard ratios of all-cause mortality and newly developed cancers with the use of aspirin or dipyridamole. Confounding factors were incorporated into the models. We also used a defined daily dose (DDD), which is recommended by the World Health Organization as a unit to measure the total amount of drugs used. The DDD was calculated according to the following formula: (total amount of drugs)/(amount of drugs in a DDD) = number of DDDs. To evaluate the duration of exposure, we calculated cumulative DDDs of aspirin and dipyridamole from the index date to the end of the observation period (December 31, 2013). We set 75 mg as the cut-off point in subgroup analysis for both aspirin and dipyridamole, which 75 mg is the lowest dose and common dosage form usually prescribed to the patients according to the local package inserts suggested for cardiovascular disease prevention with the minimal side effect.^[24,25]^ Statistical significance was set at *P* < .05. All data processing and statistical analysis were performed using SAS software version 9.4 (Cary, NC).

## 3. Results

A total of 5308 patients (2533 males and 2775 females) with type 2 diabetes mellitus were included. Table 1 shows their clinical characteristics. Baseline differences among 3 groups, 1176 no aspirin nor dipyridamole users, 1424 dipyridamole users, and 2708 aspirin users were identified. These 3 groups were noted in a history of pulmonary disease (5.4%, 8.1%, and 5.9%, respectively; *P* value = .0057), liver disease (6.6%, 5.6%, and 4.5%; *P* value = .0164), nephropathy (1.8%, 3.6%, and 2.0%; *P* value = .0032), renal disease (0.8%, 2.0%, and 1.4%; *P* value = .0336), a history of taking other antihypertension drugs (1.2%, 2.4%, and 1.1%; *P* value = .0034), insulin (47.5%, 62.3%, and 59.7%; *P* value < .0001), metformin (96.3%, 98.2%, and 96.9%; *P* value = .0067), sulfonylureas (97.8%, 99.0%, and 98.5%; *P* value = .0394), acarbose (46.6%, 57.0%, and 55.2% *P* value < .0001), thiazolidinediones (49.3%, 57.4%, and 58.2%; *P* value < .0001) and dipeptidyl peptidase-4 inhibitor (33.7%, 36.9%, and 39.5%; *P* value .0024).

**Table 1:** Baseline demographic data of study participants.

Characteristics	No. of cases	No aspirin nor dipyridamole user	Dipyridamole user	Aspirin user	
		N (%)	N (%)	N (%)	*P* value
Total	5308	1176	1424	2708	
Female	2775	587 (49.9)	807 (56.7)	1381 (51.0)	.0004
Male	2533	589 (50.1)	617 (43.3)	1327 (49.0)	.
Age (mean ± SD)	58.22 ± 11.43	54.97 ± 12.54	59.89 ± 11.11	58.76 ± 10.81	<.0001
Acute myocardial infarction	13	2 (0.2)	5 (0.4)	6 (0.2)	.6103
Congestive heart failure	28	4 (0.3)	13 (0.9)	11 (0.4)	.0615
Peripheral vascular disease	24	3 (0.3)	6 (0.4)	15 (0.6)	.4344
Cerebral vascular accident	171	30 (2.6)	50 (3.5)	91 (3.4)	.3252
Dementia	13	2 (0.2)	4 (0.3)	7 (0.3)	.8330
Pulmonary disease	339	64 (5.4)	116 (8.1)	159 (5.9)	.0057
Connective tissue disorder	22	3 (0.3)	5 (0.4)	14 (0.5)	.4603
Peptic ulcer	224	50 (4.3)	71 (5.0)	103 (3.8)	.1987
Liver disease	279	78 (6.6)	80 (5.6)	121 (4.5)	.0164
Nephropathy	125	21 (1.8)	50 (3.5)	54 (2.0)	.0032
Retinopathy	111	15 (1.3)	36 (2.5)	60 (2.2)	.0688
Neuropathy	278	55 (4.7)	73 (5.1)	150 (5.5)	.5282
Paraplegia	10	2 (0.2)	3 (0.2)	5 (0.2)	.9701
Renal disease	74	9 (0.8)	28 (2.0)	37 (1.4)	.0336
Severe liver disease	14	4 (0.3)	2 (0.1)	8 (0.3)	.5524
Human immunodeficiency virus	1	1 (0.1)	0 (0.0)	0 (0.0)	.1725
Chronic hepatitis B	19	5 (0.4)	9 (0.6)	5 (0.2)	.0663
Chronic hepatitis C	25	3 (0.3)	11 (0.8)	11 (0.4)	.1241
Duration of use Mean ± SD (yr)			3.20 ± 3.74	5.31 ± 4.23	
**Antihypertension medications**					
Angiotensin-converting enzyme inhibitors	122	17 (1.4)	39 (2.7)	66 (2.4)	.0717
Angiotensin II receptor blockers	15	3 (0.3)	4 (0.3)	8 (0.3)	.9765
Calcium channel blocker	238	33 (2.8)	83 (5.8)	122 (4.5)	.0010
Beta-blocker	88	12 (1.0)	30 (2.1)	46 (1.7)	.0945
Diuretics	47	8 (0.7)	18 (1.3)	21 (0.8)	.1956
Hydralazine	16	4 (0.3)	5 (0.4)	7 (0.3)	.8429
Alpha-blocker	41	6 (0.5)	12 (0.8)	23 (0.8)	.5077
Fibrates	49	9 (0.8)	10 (0.7)	30 (1.1)	.3517
Other antihypertension medication	78	14 (1.2)	34 (2.4)	30 (1.1)	.0034
**Antihyperglycemic medications**					
Insulin	3063	559 (47.5%)	887 (62.3%)	1617 (59.7%)	<.0001
Metformin	5155	1132 (96.3%)	1399 (98.2%)	2624 (96.9%)	.0067
Sulfonylureas	5227	1150 (97.8%)	1410 (99.0%)	2667 (98.5%)	.0394
Acarbose	2856	548 (46.6%)	812 (57.0%)	1496 (55.2%)	<.0001
Thiazolidinediones	2972	580 (49.3%)	817 (57.4%)	1575 (58.2%)	<.0001
Dipeptidyl peptidase-4 inhibitor	1990	396 (33.7%)	525 (36.9%)	1069 (39.5%)	.0024
Meglitinides	242	51 (4.3%)	60 (4.2%)	131 (4.8%)	.6044
Glucagon-like peptide 1 agonist	16	4 (.3%)	1 (.1%)	11 (.4%)	.1669

SD = standard deviation.

Table 2 shows that the patients who took dipyridamole and aspirin had a lower incidence rate of liver cancer (2.4% and 2.0%, respectively) compared to those who did not use aspirin or dipyridamole (4.0%). The aspirin users also had a lower incidence rate of bladder cancer (0.2%) compared to the dipyridamole users (0.7%) and those who did not use aspirin or dipyridamole (0.3%).

**Table 2:** Cancer risk ratio in type 2 diabetic patients with no aspirin nor dipyridamole user, dipyridamole user, and aspirin user.

	No. of cases	No aspirin nor dipyridamole user	Dipyridamole user	Aspirin user	
		N (%)	N (%)	N (%)	*P* value
Any type of cancer	536	135 (12.0)	150 (11.2)	251 (97)	.0807
All-cause mortality	496	92 (7.8)	135 (9.5)	269 (9.9)	.1134
Liver	135	46 (4.0)	34 (2.4)	55 (2.0)	.0024
Colon	43	10 (0.9)	13 (0.9)	20 (0.7)	.8253
Lung	67	17 (1.5)	16 (1.1)	34 (1.3)	.7834
Breast	35	11 (0.9)	8 (0.6)	16 (0.6)	.4144
Prostate	26	6 (0.5)	7 (0.5)	13 (0.5)	.9925
Rectum	36	9 (0.8)	12 (0.8)	15 (0.6)	.5197
Stomach	26	8 (0.7)	7 (0.5)	11 (0.4)	.5305
Bladder	18	3 (0.3)	10 (0.7)	5 (0.2)	.0207
Cervix	16	3 (0.3)	4 (0.3)	9 (0.3)	.9088
Kidney	24	4 (0.3)	11 (0.8)	9 (0.3)	.1069
Lymphoma	7	4 (0.3)	2 (0.1)	1 (0.0)	.0567
Esophagus	10	5 (0.4)	2 (0.1)	3 (0.1)	.1030
Pancreas	13	4 (0.3)	2 (0.1)	7 (0.3)	.5781
Bile duct	6	1 (0.1)	0 (0.0)	5 (0.2)	.2316
Larynx	3	1 (0.1)	0 (0.0)	2 (0.1)	.5715
Ovary	3	0 (0.0)	1 (0.1)	2 (0.1)	.6518
Thyroid	7	1 (0.1)	2 (0.1)	4 (0.1)	.8799
Leukemia	1	0 (0.0)	1 (0.1)	0 (0.0)	.2556

After adjusting for the confounding factors in Table 1, Table 3 shows the effects of aspirin and dipyridamole on the risk of any type cancer, and liver, colon, lung, bladder cancer, and lymphoma. Dipyridamole significantly decreased the risk of liver cancer (risk ratio [RR] = 0.51, 95% confidence interval [CI] = 0.32–0.80). Aspirin significantly decreased any type of cancer, liver cancer, and lymphoma (RR = 0.72, 95% CI = 0.59–0.89; RR = 0.46, 95% CI = 0.31–0.69; and RR = 0.11, 95% CI = 0.01–0.96), respectively.

**Table 3:** Risk ratios for any types of cancer, lymphoma and liver, colon, lung, and bladder cancer in type 2 diabetic patients with dipyridamole or aspirin use.

	Dipyridamole		Aspirin	
	AHR (95% CI)	*P* value	AHR (95% CI)	*P* value
Any types of cancer	0.82 (0.65–1.04)	.1088	0.72 (0.59–0.89)	.0026
All-cause mortality	0.94 (0.72–1.23)	.6595	1.08 (0.85–1.37)	.5203
Liver cancer	0.51 (0.32–0.80)	.0037	0.46 (0.31–0.69)	.0001
Colon cancer	0.82 (0.36–1.90)	.6463	0.67 (0.31–1.44)	.3004
Lung cancer	0.68 (0.34–1.36)	.2803	0.77 (0.43–1.38)	.3734
Bladder cancer	2.14 (0.59–7.85)	.2501	0.53 (0.13–2.24)	.3899
Lymphoma cancer	0.35 (0.06–1.95)	.2309	0.11 (0.01–0.96)	.0455

All model adjusted variable in Table 1.

AHR = adjusted hazard ratio, CI = confidence interval.

We further analyzed the effects of low- and high-dose dipyridamole and aspirin on risk of cancer with 75 mg as the cut-off point (Table 4). After adjusting for multiple covariates, a high dose of dipyridamole decreased the risk of liver cancer (RR = 0.61, 95% CI = 0.38–0.99; and RR = 0.28, 95% CI = 0.12–0.66, respectively), any type of cancer (RR = 0.79, 95% CI = 0.64–0.98; and RR = 0.49, 95% CI = 0.34–0.70), and the incidence of liver cancer (RR = 0.56, 95% CI = 0.37–0.83; and RR = 0.14, 95% CI = 0.05–0.39). With regard to all-cause mortality, low-dose aspirin had a RR of 1.33 (95% CI = 1.04–1.69), but high-dose aspirin had a RR of 0.28 (95% CI = 0.16–0.47).

**Table 4:** Risk ratios for any type, liver, colon, lung, bladder cancer, and lymphoma in type 2 diabetic patients with low and high doses of dipyridamole and aspirin.

	Dipyridamole				Aspirin			
	Low dose (≤75 mg)		High dose (>75 mg)		Low dose (≤75 mg)		High dose (>75 mg)	
	HR (95% CI)	*P* value	HR (95% CI)	*P* value	HR (95% CI)	*P* value	HR (95% CI)	*P* value
Any types of cancer	0.87 (0.67–1.12)	.2765	0.74 (0.52–1.04)	.0841	0.79 (0.64–0.98)	.0347	0.49 (0.34–0.70)	.0001
All-cause mortality	0.98 (0.73–1.31)	.8852	0.88 (0.61–1.26)	.4791	1.33 (1.04–1.69)	.0213	0.28 (0.16–0.47)	<.0001
Liver cancer	0.61 (0.38–0.99)	.049	0.28 (0.12–0.66)	.0038	0.56 (0.37–0.83)	.0046	0.14 (0.05–0.39)	.0002
Colon cancer	0.77 (0.30–1.99)	.5934	0.91 (0.31–2.71)	.8707	068 (0.31–1.52)	.3468	0.62 (0.19–1.97)	.4124
Lung cancer	0.84 (0.41–1.75)	.6438	0.38 (0.11–1.29)	.1207	0.81 (0.44–1.49)	.4985	0.61 (0.24–1.55)	.2969
Bladder cancer	1.85 (0.46–7.54)	.389	2.75 (0.60–12.5)	.192	0.28 (0.05–1.66)	.1592	0.28 (0.05–1.66)	.1592
Lymphoma cancer	0.24 (0.03–2.23)	.2103	0.61 (0.07–5.58)	.6626	0.14 (0.02–1.29)	.0826	-	-

All model adjusted variable in Table 1.

CI = confidence interval, HR = hazard ratio.

## 4. Discussion

In this study, both low- and high-dose dipyridamole and aspirin decreased the risk of liver cancer in patients with type 2 diabetes. Low- and high-dose aspirin also decreased the risk of any type of cancer, and high-dose aspirin decreased the risk of all-cause mortality.

Hepatocellular carcinoma is one of the most prevalent cancers and the third most common cause of cancer death worldwide,^[26]^ and the incidence continue to rise yearly. Liver cirrhosis, hepatitis B and C viral infection, steatosis, and alcohol intake are risk factors for hepatocellular carcinoma. Relevant risk factors include being overweight and having metabolic syndrome. Liver cirrhosis is most associated with hepatocellular carcinoma.^[27–29]^

In Taiwan, chronic hepatitis viral infection is the underlying etiology for more than 90% of all cases of hepatocellular carcinoma, and 3.50 and 1.70 million people have been reported to be hepatitis B viral and hepatitis C viral carriers, respectively.^[30]^ The crude mortality rate of hepatocellular carcinoma in Taiwan has been reported to be 30.21 per 100,000 person-years, and hepatocellular carcinoma is the leading cause of cancer-related mortality in males and the second leading cause in females.^[30,31]^

Reducing risk factors, early diagnosis, and treatment can decrease the incidence of cancer-related deaths. Hence, screening for hepatocellular carcinoma is recommended for high-risk groups.^[32,33]^ Steatosis is one of the risk factors for hepatocellular carcinoma and is often found in a patient with diabetes. In addition, nonalcoholic fatty liver disease is also prevalent among patients with diabetes or obesity, and even more common in patients with both. Over 70% of patients with obesity and type 2 diabetes have nonalcoholic fatty liver disease.^[34]^ Other factors associated with the risk of hepatocellular carcinoma in patients with diabetes include hepatitis B viral and hepatitis C viral infections, both of which occur more frequently in diabetic subjects compared to those without diabetes.^[35,36]^

Cancer is one of the most common causes of mortality in Taiwan and worldwide. Studies have reported an association between the regular use of aspirin and reduced risks of colorectal, esophageal, breast, lung, prostate, skin,^[37–40]^ and liver cancer.^[41]^ Aspirin has been shown to prevent cancer cell growth and lead to apoptosis in several colon cancer cells and tumor model studies.^[42,43]^ Cancer patients have more activated platelet function, and this is related to disease progression and metastasis.^[44]^

The inhibition of COX has been shown to be responsible for the antithrombotic and anti-inflammatory effects of aspirin. In addition, aspirin and its primary metabolite, salicylate, have been shown to reduce the expression of COX-2, thereby decreasing the synthesis of pro-inflammatory prostaglandins.^[45]^ Moreover, aspirin has been shown to be a considerably more effective inhibitor of COX-1 in anucleate platelets than COX-2 in monocytes, and this can cause long-lasting thromboxane A2-dependent platelet dysfunction.^[46]^ The inhibition of COX has also been shown to increase levels of arachidonic acid, and this can then result in the conversion of sphingomyelin to ceramide, which is a mediator of apoptosis.

For liver cancer, a pooled analysis of 2 large, prospective cohorts (the Nurses’ Health Study and the Health Professionals Follow-up Study) reported the use of aspirin and the incidence of hepatocellular carcinoma in 133,371 healthcare professionals over more than 26 years. The results showed that regular, long-term use of aspirin was associated with a statistically significant 49% reduction (adjusted hazard ratio = 0.51, 95% CI = 0.34–0.77) in the risk of hepatocellular carcinoma compared to those who did not take aspirin regularly or at all, and that this effect was apparent after 5 or more years of use.^[46]^ The anti-inflammatory effect of aspirin may delay the progression of fibrosis or directly reduce the risk of hepatocellular carcinoma.^[47]^ A previous cohort study conducted from September 2007 to September 2017 including 227 patients with hepatocellular carcinoma reported that a total of 208 (91.63%) of the patients had died, and the 5-year survival rate was only 8.37% with a median overall survival rate of 12.1 months.^[48]^

The exact mechanisms underlying the anticancer effects of aspirin are unknown. Although several mechanisms have been proposed, including those affecting cellular signaling, enzyme activity, transcription factors, and mitochondrial function.^[49]^

One previous review reported that the effect of aspirin in inhibiting platelet activation may mediate the cancer-preventive and cardio-protective effects of low-dose aspirin. Secondary analysis of cardiovascular trials has shown that daily low-dose aspirin (75–100 mg daily) may also reduce the incidence of all cancers, although the magnitude of the potential benefit is unknown. However, even a 10% reduction in the overall incidence of cancer could substantially increase the indications for prophylactic daily low-dose aspirin treatment.^[42]^ The dose of aspirin varies according to the condition being treated, such as 1.2 g for anti-inflammatory purposes, 325 to 600 mg for analgesic purposes, and 75 mg for antiplatelet purposes.^[43]^ However, a recently published meta-analysis that included 34 trials of daily aspirin treatment at various doses found that daily aspirin at doses of 75 mg and above may lower both the overall incidence of cancer and cancer-related deaths.^[14]^ In addition, the meta-analysis also found that the rate of cancer mortality was reduced during the more than 5 years of follow-up; and that the degree of the benefits was not higher in the patients with a daily dose higher than 75 to 100 mg.^[14]^ Moreover, several long-term randomized trials comparing patients receiving daily aspirin treatment and controls with regard to the prevention of vascular events reported that those who received aspirin had lower incidence and mortality rates of colorectal cancer after 8 to 10 years^[50,51]^ and reduced mortality rates of several other common cancers after 5 to 15 years.^[52]^

Several studies have reported that doses of aspirin ranging from 81 to 325 mg taken over prolonged periods can decrease the incidence and mortality associated with colorectal cancer.^[14,43,50]^ But in populations at average risk, this benefit does not outweigh the potential harm from aspirin-induced bleeding.^[42]^ Aspirin is not currently recommended as a prophylaxis treatment for cancer due to its adverse effects, including the risk of bleeding.

The mechanism of the antiplatelet agent dipyridamole includes increasing the concentration of intracellular cyclic adenosine monophosphate, inhibiting changes in the shape of platelets. This increase in cyclic adenosine monophosphate concentration has been reported to be due to the inhibition of phosphodiesterase and/or blocking adenosine uptake.^[53]^ Dipyridamole is given either alone or in combination with aspirin to manage stroke and myocardial infarction. In addition, dipyridamole may be given as a modified-release preparation at a dose of 200 mg twice daily for the secondary prevention of stroke or transient ischemic attack. The intravenous administration of dipyridamole led to obvious coronary vasodilation. Thus, it can be used as stress test for ischemic heart disease.^[54]^

Few studies have investigated the association between dipyridamole and malignancy. In in vivo models, the intraperitoneal administration of dipyridamole has been shown to significantly reduce primary tumor growth and metastasis. Moreover, dipyridamole has been shown to significantly decrease macrophage infiltration associated with tumors and suppressor cells derived from myeloid cells in primary tumors, and also to decrease levels of inflammatory cytokines in the sera of treated mice.^[55]^

The main strengths of this study are that it is a large cohort study including 5308 diabetic patients with a long follow-up period. In addition, the diagnoses of cancer and diabetes are likely to be accurate as we used data from the NHIRD. The NHIRD is population-based and highly representative, and thus there is little possibility of recall and selection bias. There are also several limitations to this study. First, we did not have data on the duration, dosage, and adherence to aspirin and dipyridamole, nor an accurate measurement of aspirin or dipyridamole serum levels to investigate the effect on cancer prevention, even though several studies have shown that long-term aspirin usage can prevent malignant diseases. Second, because of the limited data of NHIRD, we were not able to review associated risk factors for cancer, such as smoking, alcohol drinking, the duration of diabetes mellitus, family history of malignancies, and other socioeconomic characteristics, or major modifiable determinants of diabetes and insulin resistance, such as obesity and body mass index. Further, we only selected patients with catastrophic illness cards to ensure the accuracy of the diagnosis of cancer but not be able to identify further the histopathology type of different cancers, and we may have missed some patients who had been waiting for a pathological diagnosis and had not yet received catastrophic cards. Finally, due to diabetes causing many complications such as coronary heart disease, cerebral vascular disease, nephropathy, retinopathy, and neuropathy, diabetic patients may take more medications than other populations. However, we only evaluate the effect of some medications.

## 5. Conclusions

Aspirin can decrease the risk of several types of cancer; however, diabetes reduces this protective effect of aspirin. Nevertheless, aspirin still had a protective effect against many types of cancer and liver cancer, and dipyridamole also decreased the risk of liver cancer in the diabetic patients in this study. Therefore, both low and high doses of aspirin and dipyridamole may decrease the risk of liver cancer in patients with type 2 diabetes. Even there are still unresolved questions about the treatment regimen of aspirin and dipyridamole needed to prevent cancer. The risk of cancer is higher in patients with diabetes. As the population is aging and the incidence of diabetes is increasing, we suggest patients with diabetes should accept appropriate cancer screening.

As the number of diabetic patients and the prevalence of malignancy is increasing worldwide, preventing the occurrence of malignancy is important. Our results found that aspirin and dipyridamole can prevent liver cancer, and hepatocellular carcinoma is still highly prevalent in Taiwan. Therefore, aspirin and dipyridamole may be used to decrease the risk of liver cancer in patients with type 2 diabetes in the future.

## Author contributions

**Conceptualization:** Hsing-Yi Huang.

**Data curation:** Wei-Hao Hsu, Mei-Yueh Lee.

**Formal analysis:** Hsiu-Ling Liang.

**Funding acquisition:** Wei-Hao Hsu.

**Investigation:** Ya-Wen Sung.

**Resources:** Hsing-Yi Huang.

**Software:** Hsiu-Ling Liang.

**Supervision:** Ya-Wen Sung.

**Writing – original draft:** Tz-Wen Lu.

**Writing – review & editing:** Hsing-Yi Huang.

## Acknowledgments

The authors thank Yu-Ting Huang of the Statistical Analysis Laboratory, Department of Medical Research, Kaohsiung Medical University Hospital, Kaohsiung Medical University.
